# Unpredictable environments enhance inhibitory control in pheasants

**DOI:** 10.1007/s10071-019-01302-0

**Published:** 2019-08-30

**Authors:** Jayden O. van Horik, Christine E. Beardsworth, Philippa R. Laker, Ellis J.G. Langley, Mark A. Whiteside, Joah R. Madden

**Affiliations:** Centre for Research in Animal Behaviour, Washington Singer Laboratories, Psychology, College of Life and Environmental Sciences, University of Exeter, Exeter, EX4 4QG UK

**Keywords:** Executive function, Response inhibition, Impulsivity

## Abstract

The ability to control impulsive actions is an important executive function that is central to the self-regulation of behaviours and, in humans, can have important implications for mental and physical health. One key factor that promotes individual differences in inhibitory control (IC) is the predictability of environmental information experienced during development (i.e. reliability of resources and social trust). However, environmental predictability can also influence motivational and other cognitive abilities, which may therefore confound interpretations of the mechanisms underlying IC. We investigated the role of environmental predictability, food motivation and cognition on IC. We reared pheasant chicks, *Phasianus colchicus*, under standardised conditions, in which birds experienced environments that differed in their spatial predictability. We systematically manipulated spatial predictability during their first 8 weeks of life, by either moving partitions daily to random locations (unpredictable environment) or leaving them in fixed locations (predictable environment). We assessed motivation by presenting pheasants with two different foraging tasks that measured their dietary breadth and persistence to acquire inaccessible food rewards, as well as recording their latencies to acquire a freely available baseline worm positioned adjacent to each test apparatus, their body condition (mass/tarsus^3^) and sex. We assessed cognitive performance by presenting each bird with an 80-trial binary colour discrimination task. IC was assessed using a transparent detour apparatus, which required subjects to inhibit prepotent attempts to directly acquire a visible reward through the barrier and instead detour around a barrier. We found greater capacities for IC in pheasants that were reared in spatially unpredictable environments compared to those reared in predictable environments. While IC was unrelated to individual differences in cognitive performance on the colour discrimination task or motivational measures, we found that environmental predictability had differential effects on sex. Males reared in an unpredictable environment, and all females regardless of their rearing environment, were less persistent than males reared in a predictable environment. Our findings, therefore, suggest that an individual’s developmental experience can influence their performance on IC tasks.

## Introduction

Executive functions regulate an individual’s thoughts, actions and behaviours (Miyake and Friedman 2012). A central executive function is inhibitory control (IC), the ability to suppress urges, resist temptations and delay gratification (Diamond 2013). In human and non-human animals, individuals differ in their capacities for IC (Friedman et al. 2008; Friedman and Miyake 2017; van Horik et al. 2018a). For humans, environmental and social experiences during early development may influence long-term individual differences in IC (Diamond and Lee 2011), which leads to subsequent differences in personal well-being during adulthood (Moffitt et al. 2011). Children show short-term impairments in IC after experiencing unreliable, rather than reliable, information about the availability of resources (Kidd et al. 2013) and when interacting with untrustworthy, rather than trustworthy, characters (Michaelson et al. 2013), which, if reinforced, may also lead to long-term individual differences in IC. Impulsive behaviours may, however, be considered dysfunctional or functional depending on the environmental and social factors that an individual experiences (Dickman 1990; Gullo and Dawe 2008). Low IC, for example, might be expected to result from biological adaptations to harsh and unpredictable environments, where people prefer immediate over delayed rewards (Frankenhuis et al. 2016). An individual’s experiences and beliefs about the predictability of their social and physical world are, therefore, likely to influence their capacities for IC.

Accurate assessments of the environmental effects that influence IC may also be confounded by corresponding changes in other, unrelated, cognitive or motivational processes. For example, complex and unpredictable environments, in which an individual is exposed to a large number of different challenges during their lifespan, can bolster more general capacities for learning, demonstrated in both natural systems (Menzel and Muller 1996) and predictive models (Dridi and Lehmann 2016; Niv et al. 2002). Consequently, differences in IC may result from general changes in cognitive ability arising from the unpredictable environment, rather than a direct response to environmental effects. Environments characterised by uncertainty may also influence an individual’s motivation, for example, through associated food-seeking behaviours, which indirectly alter the expression of IC (Shaw 2017). The relationship between motivational responses and uncertainty has been shown both mechanistically, with stronger responses to a conditioned stimulus that unreliably predicts food delivery, and functionally, in animals that seek, consume and/or hoard more food when access to that resource is unpredictable (Anselme and Güntürkün 2018). Consequently, environmental uncertainty may also influence both motivational and cognitive processes that confound accurate interpretations of how the environment shapes IC. Understanding the factors that shape individual differences in IC is important because, at least in humans, effective IC predicts a suite of favourable traits, such as improved educational and financial success, mental and physical health, interpersonal skills and relationships, and may prevent unfavourable traits, such as binge eating, drug abuse and criminal behaviour (Moffitt et al. 2011; Tangney et al. 2004). However, because of the confounding or complementary influences of multiple processes (such as broader cognitive abilities and/or motivation) that may moderate developmental influences in unpredictable ways, the critical elements that shape IC in humans remain obscured.

The developmental processes that drive individual differences in IC may be revealed by using animal models that can be experimentally manipulated to test the influence of particular factors. One behavioural assay of IC that is common to both human and non-human animals is the suppression of motor actions, which can be assayed using a detour task (Diamond 1990; Kabadayi et al. 2018; MacLean et al. 2014). This task requires subjects to inhibit attempts to acquire a clearly visible, but inaccessible food reward that is positioned behind a transparent barrier and instead move away from the visible reward and detour around the obstacle to access the food (i.e. response inhibition; Nigg 2017). An individual exhibiting good IC will make fewer attempts to directly access the reward through the transparent barrier and instead inhibit such unrewarded attempts and rapidly switch to an alternative, indirect method or route. Performances of animals on detour tasks have been related to absolute brain size (MacLean et al. 2014), but support for this relationship remains contentious (Jelbert et al. 2016; Kabadayi et al. 2017a, b; Lucon-Xiccato et al. 2017; van Horik et al. 2018a; Völter et al. 2018). In animals, individual performance on IC tasks is also predicted by non-cognitive traits such as high food motivation (van Horik et al. 2018a), low body condition (Shaw 2017), high arousal (Bray et al. 2015), environmental enrichment (Clarke et al. 1951), fearfulness (Regolin et al. 1995) and prior experience with transparent objects (van Horik et al. 2018a). While detour tasks are relatively simple to deploy, and have a general applicability across species (MacLean et al. 2014), it remains necessary to illuminate the putative cognitive processes that mediate performances on detour tasks, while controlling for the non-cognitive, motivational traits that confound the accuracy of measures on these tasks.

We used a novel animal model to investigate the role of environmental predictability, motivation and general learning ability on IC. We reared pheasant chicks, *Phasianus colchicus*, in environments that we made either spatially unpredictable or predictable by moving (or not moving) partitions on a daily basis for the first 8 weeks of their life. We then assayed colour discrimination learning and multiple motivational traits of birds, to determine whether these traits were simultaneously altered by our environmental manipulation, and thus whether they also had differential influences on IC. To assess capacities for IC, each individual experienced one of two different detour tasks, in which they were required to inhibit pecking at a visible but inaccessible food item placed behind a transparent barrier, or inside a transparent cylinder (van Horik et al. 2018a). Measures of learning, as a general proxy for cognitive ability in pheasants, were obtained from a reinforcement learning task involving 80 binary discriminations between a rewarded and unrewarded colour cue. Such tasks have previously shown to reflect robust individual differences in learning ability (van Horik et al. 2018b, c, 2019). Motivation was assessed using each individual’s body condition (mass/tarsus^3^), their latencies to acquire a freely available baseline worm placed adjacent to the test apparatus, and their performance on two additional tasks. The first task measured each individual’s persistence in trying to obtain a visible, but inaccessible, food reward and the second task measured their dietary breadth using a free-choice task of familiar food items. As pheasants are sexually dimorphic (Hill and Robertson 1988; Whiteside et al. 2018), body mass and body condition likely differ between sexes and so we also included sex in the analyses to account for this influence on motivation. If the predictability of the environment has a consistent, and general, effect on IC across social and physical contexts and species then based on social manipulations of the reliability of resource availability, as demonstrated in children (e.g. Kidd et al. 2013), we expected that birds reared in predictable environments would show greater IC (making fewer pecks at a barrier) than birds reared in unpredictable environments. It is possible that our manipulation of environmental predictability also affected other capacities for learning or motivational traits that in turn influenced performances on detour tasks; therefore, we simultaneously tested whether these other factors also differed following our manipulation. Finally, we tested whether, between individuals, any of these potential cognitive and non-cognitive factors provided a better explanation of differences in IC.

## Methods

### Subjects and housing

One hundred and seventy-nine pheasant chicks were hatched on the same day from an incubator, randomly assigned to 3 groups of 45 and 1 group of 44 in replicated enclosures and reared between 22 May 2017 and 28 July 2017. Birds were individually marked using numbered wing tags, fed on commercial pheasant feed (Keepers’ Choice) and supplied with water ad libitum. Birds were housed in 2 m × 2 m heated pens (indoor hut) for the first 2 weeks of life. They had access to unheated but covered outdoor enclosure (night shelter) of 1 m × 4 m for the next week. For the final 7 weeks of rearing, each group had additional access to separate 4 m × 12 m outdoor runs.

### Procedure

Birds were habituated to human observation from 1 day old. Shaping procedures, using mealworm rewards, were adopted to habituate subjects to an experimental chamber (0.75 m × 0.75 m) that was positioned adjacent to their pens. During testing, an experimenter opened a sliding door that allowed the birds to individually enter the experimental chamber, where the subject’s performance was observed. All birds were tested while visually isolated from others. After testing, subjects were released into the outdoor run. Subjects that failed to engage with the tasks within a predetermined time (described for each task below) were released from the experimental chamber and excluded from analyses.

### Manipulating environmental predictability

All birds were exposed to barriers for 8 weeks prior to assays of their IC being obtained. Each enclosure contained nine opaque partitions constructed from black plastic and wood. Three partitions were placed inside the indoor hut (70 cm wide × 40 cm high), three were placed in the night shelter (70 cm wide × 40 cm high) and the remaining three partitions (100 cm wide × 80 cm high) were placed in the outdoor run. In two of the four pens, all partitions were moved daily to a randomly determined location that was matched between pens (unpredictable condition). In the remaining two pens, the partition locations remained consistent (predictable condition). All pens otherwise had equal experiences including (sham) disturbance, in which experimenters entered the predictable condition pens and repositioned the barriers into identical locations.

### Assessing inhibitory control

We presented pheasant chicks with one of two different detour tasks involving a cylinder or barrier. Birds from two pens (one predictable, one unpredictable) experienced the cylinder task and birds from the remaining two pens experienced the barrier task. Performances of pheasants on both barrier and cylinder tasks, presented in a counter-balanced order, alongside their estimates of repeatability are reported in van Horik et al. (2018a). In both tasks, birds first participated in four training trials on an opaque training apparatus to ensure that they could access a reward that was placed either inside the cylinder or behind the barrier before participating in a single test trial on an identical transparent variant of the apparatus. As the mealworm reward was clearly visible during the test trial, subjects had to inhibit their prepotent attempts to acquire the reward directly through the transparent cylinder or barrier and instead detour around the obstacle to access the reward, as they had previously learned during the opaque training trials. We recorded the number of pecks (incorrect attempts) each individual directed towards the transparent barrier before acquiring the mealworm reward as a measure of their IC. All pecks made at the transparent cylinder/barrier were targeted towards the food reward and hence considered a failure of motor inhibition. Birds that failed to interact with the apparatus within 240 s were released from the testing chamber and excluded all subsequent analyses. Birds participated in their training trials on 13 July 2017 (53 days old) and their test trials on 14 July 2017. Further details of procedures and apparatus can be found in van Horik et al. (2018a).

### Assessing learning: colour discrimination task

Subjects were shaped to peck through a layer of crepe paper and retrieve a mealworm reward concealed in a well. During testing, a pair of wells, each 2 cm diameter, 1.8 cm deep, and 2 cm apart, was presented, covered with crepe paper to conceal its contents. One well was encircled by a blue cue and contained a single mealworm reward. The other well, encircled by a green cue, was unrewarded and blocked by a solid barrier to impose a moderate cost on birds attempting to peck through the crepe paper. The location of the rewarded and unrewarded wells was pseudorandomised across trials so that the same cue was not presented more than three times in a row at the same location. Each subject received eight 10-trial sessions between 19 and 22 June 2017 (29 and 32 days old), comprising one session in the morning between 08:00 and 10:00, and one in the afternoon between 13:00 and 15:00. During each trial, birds were required to make a binary choice between the blue and green colour cues. A correct choice was scored if subjects pecked a rewarded well and an incorrect choice was scored if subjects pecked an unrewarded well. If the bird made a correct choice, it was allowed to eat the reward before a new pair of wells was presented. If the bird made a wrong choice, the pair of wells was removed and replaced with a new pair. Subjects were released from the test chamber once they completed ten trials or after 120 s if they failed to complete ten trials. We generated learning curves to provide measures of individual performance across trials. Learning curve coefficients were derived from the probability of making a correct or incorrect choice per trial, after fitting a sigmoid curve to the binary choice data using R (R Development Core Team 2014). We used the predicted trial number when the curve exceeded an 80% probability of the bird making a correct choice as our performance measure (see van Horik et al. 2018b). Learning curves accounted for individuals with a strong positive bias, as these birds showed poor improvement in performance. At the population level, all pheasants showed an improvement in their performances on the colour learning task (Paired *t* test: *t*_1,116_ = 17.875, *P* < 0.0001), making approximately 40% correct choices on their first 10 trials (mean = 4.195 ± 0.227 SEM) and reaching > 80% correct choices on their final 10 trials (mean = 8.036 ± 0.168 SEM).

### Assessing motivation

We used five separate measures that we considered to account for different aspects of an individual’s motivation. First, we took a single measure of how long each bird took from entering the experimental chamber to acquire a freely available mealworm placed in front of the IC apparatus, termed the *baseline worm latency*. This measure provided an indication of an individual’s motivation to acquire a desirable food item. Second, we recorded how long a bird would persist in trying to access visible, but inaccessible, food items, termed the *persistence task*. For this, a transparent Petridish (8 cm diameter) with its lid glued shut was fastened horizontally to a white base (20 × 20 cm) and contained approximately 70 live, but inaccessible, mealworms. We recorded the number of pecks each bird made towards the apparatus within 1 min as a measure of their persistence. Third, we assessed the dietary breadth of each bird as a measure of motivation to take novel food items in the *dietary breath Task*. Birds were familiarised with an ad libitum supply of commercial parrot food, containing a variety (> 10) of different food items (i.e. seeds, dried fruits, chilli peppers, different coloured kibble, for 7 days prior to testing. During testing, we presented birds with a fixed array of ten different food items (two examples of each food type). We recorded how many different food items that each bird sampled within a single 2-min trial. Birds were tested individually on these last two tasks on the 19th July 2017 (59 days old). Fourth, we collected a morphological indicator of *body condition*. Two days after testing had ceased, when birds were 70 days old, we recorded each individuals’ mass, using a spring balance scale (Slater Super Samsom–precision 5 g), and tarsus length, using a calliper (precision 0.1 mm), to determine their body condition (mass/tarsus^3^). Birds in poor (low scores) body condition were considered to be more food motivated than birds in good (high scores) body condition. Finally, as males are larger than females and differences in growth rates may result in differences in food motivation, we also included each individual’s *sex*. We visually identified the sex of individuals after they became sexually dimorphic at 10 weeks of age.

### Inclusion/exclusion of subjects for analyses

To ensure that experience on each task was standardised across subjects, we only included birds that participated on all trials for all tasks. A total of 117 birds, including 51 from the unpredictable condition (28 male; 23 female) and 66 from the predictable condition (41 male; 25 female), participated in all trials and were included in the analyses. Hence, all birds included in this study participated in 4 opaque IC training trials, 1 transparent IC test trial, 80 colour learning trials, 1 persistence trial and 1 dietary breadth trial. Birds that failed to acquire the freely available mealworm (used in the *baseline worm task*), made no pecks towards the apparatus, or did not access the mealworm rewards placed behind the transparent obstacles were considered too neophobic to obtain accurate IC measures and were hence excluded from analyses.

### Statistical analysis

First, we tested whether our manipulation of environmental predictability, a general cognitive ability, motivational traits, or sex best predicted performances on the IC task. As birds participated on two different IC tasks (barrier and cylinder), pecks and baseline worm latencies within each task were transformed to Z-scores so that the distributions within each task were comparable and performances across tasks could be compared. We included the following predictor variables: (1) environmental predictability (unpredictable or predictable); (2) baseline worm latency; (3) persistence; (4) dietary breadth; (5) body condition (mass/tarsus^3^); (6) colour learning; (7) sex and (7) IC task (cylinder or barrier). We used general linear models (GLM) with a Gaussian distribution of variance in R version 1.1.383 (R Development Core Team 2014) using the lme4 package (Bates et al. 2015) to assess what variables best predicted IC performance. All predictor variables and interactions between environment:task, environment:sex, sex:task, environment:task:sex, were included in the full model (Table 1). We then dropped terms to determine the best fitting model with the lowest AIC values (Table 2) following Burnham and Anderson (2002). We used a GLM, with the following predictor variables: baseline worm latency; persistence; dietary breadth; and sex, to test whether body condition predicted food motivation. Second, we tested whether cognitive and motivational measures differed between our experimental manipulations of environmental predictability. Cognitive measures included IC, indicated by the number of unrewarded pecks made at the apparatus, and a more general ability involving colour discrimination learning. Assays of motivation included: baseline worm acquisition latencies, persistence in attempts to acquire an inaccessible reward, dietary breadth and body condition. We used multivariate ANOVA (MANOVA) in SPSS (IBM Corp 2013) to assess whether these cognitive and motivational measures differed between the two environmental treatments or sex. Interactions between environmental treatment and sex were included for each cognitive and motivational measure. To assess whether pheasants learned the colour discrimination task, at the population level, we used a paired *t* test in SPSS and compared the number of correct choices individuals made in their first and last 10 trials. Finally, we used a Chi-squared test in SPSS to determine whether participation differed between the two environmental treatments.

**Table 1:** Full GLM and test statistics of all predictor variables that were considered to influence IC

Full model	Estimate	SE	*t*	*P*
Environment (predictable)	0.687	0.336	2.041	0.044*
Task (cylinder)	0.298	0.470	0.634	0.528
Sex (male)	− 0.198	0.349	− 0.567	0.572
Baseline worm	− 0.007	0.013	− 0.558	0.578
Persistence	0.006	0.004	1.583	0.117
Dietary breadth	0.031	0.031	1.019	0.310
Body condition	0.131	0.120	1.193	0.236
Colour learning	− 0.090	1.103	− 0.082	0.935
Environment:task	− 0.319	0.640	− 0.498	0.619
Environment:sex	− 0.196	0.468	− 0.418	0.677
Sex:task	0.052	0.607	0.085	0.933
Environment:sex:task	− 0.167	0.810	− 0.206	0.837

AIC = 340

Significant variables are denoted by *

**Table 2:** Best fitting (minimal) GLM of variables and test statistics retained after model selection

Minimal model	Estimate	SE	*t*	*P*
Environment (predictable)	0.458	0.187	2.450	0.016*
Persistence	0.006	0.004	1.596	0.113

AIC = 326.4

Significant variables are denoted by *

### Ethics

All work was approved and conducted under Home Office licence PPL 30/3204 and also approved by the University of Exeter Animal Welfare Ethical Review Board.

## Results

1. Do cognitive, motivational or environmental measures best predict IC performance?

Environmental treatment was the best predictor of IC performance, with birds reared in the unpredictable environment making fewer pecks, and hence showing greater IC, than birds reared in the predictable environment (Tables 1 and 2). We found no evidence to suggest that IC capacities were driven by differences in our measures of general learning ability (colour learning), motivation (baseline worm latency, dietary breadth, or body condition), IC task (barrier or cylinder) or sex (Tables 1 and 2).

2. Do differences in environmental treatments or sex influence cognitive or motivational measures?

**Fig. 1:**
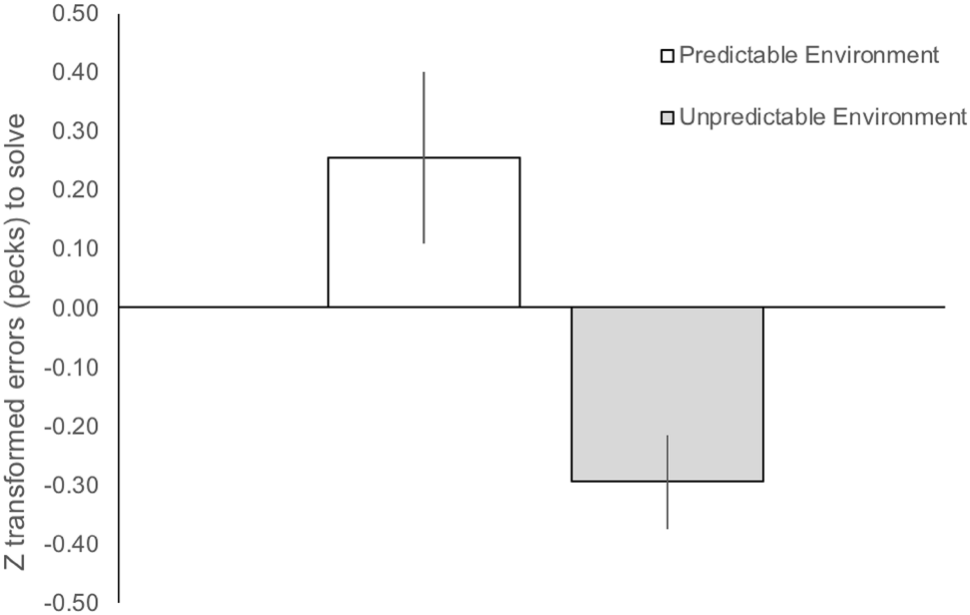
Inhibitory control performance for birds reared in either a spatially predictable or unpredictable environment. Errors (pecks) to solve a barrier or cylinder were standardised (mean centred *Z* scores ± SEM) and combined for both tasks

**Fig. 2:**
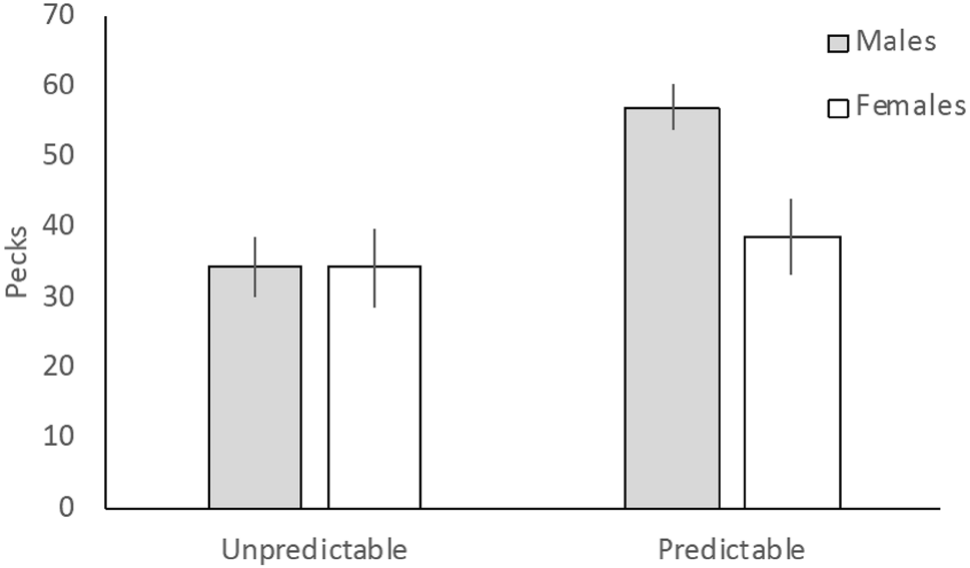
The effect of environmental predictability and sex on persistence (number of pecks) to acquire a visible but inaccessible mealworm reward (means ± SEM)

Pheasants reared in a spatially unpredictable environment made fewer errors to solve the IC task compared to pheasants reared in a spatially predictable environment (untransformed pecks: unpredictable mean = 22.255 ± 2.821 SEM; predictable mean = 38.970 ± 4.922 SEM; Table 3; Fig. 1). There was also a significant interaction between environment and sex for persistence, with males reared in the predictable environment showing greater persistence than females in the predictable environment and both sexes reared in the unpredictable environment (Fig. 2).

**Table 3:** MANOVA output of motivational and cognitive measures for environmental treatment and sex and environmental treatment:sex interactions

	Environment	Sex	Environment*Sex
Inhibitory control	*F* = 9.334, *P* = 0.003*	*F* = 0.222, *P* = 0.638	*F* = 0.036, *P* = 0.850
Colour learning	*F* = 2.976, *P* = 0.087	*F* = 0.356, *P* = 0.552	*F* = 0.117, *P* = 0.733
Baseline worm	*F* = 2.406, *P* = 0.124	*F* = 0.708, *P* = 0.402	*F* = 2.471, *P* = 0.119
Persistence	n/a	n/a	*F* = 3.989, *P* = 0.048*
Dietary breadth	*F* = 0.469, *P* = 0.495	*F* = 0.120, *P* = 0.729	*F* = 3.855, *P* = 0.052
Body condition	*F* = 0.209, *P* = 0.649	*F* = 42.992, *P* < 0.001*	*F* = 2.755, *P* = 0.100

Pecks, our measures of IC, obtained from two detour tasks (cylinder and barrier) and baseline worm latencies were transformed into *Z*-scores so that performances between the barrier and cylinder tasks were comparable and could, therefore, be amalgamated for analyses. Colour learning was derived from learning curves

DF = 1, 113

Significant variables are denoted by *

There were no differences in z-transformed baseline worm acquisition latencies, dietary breadth, body condition or colour learning between the spatially unpredictable and spatially predictable environmental treatments (Table 3). Although males had a larger body condition index than females (males mean = 12.345 ± 0.109 SEM; females mean = 11.248 ± 0.117 SEM; Table 3), no other differences were observed between sexes. Body condition was not predicted by any of our other motivational measures (baseline worm: estimate = 0.006; SE = 0.111; *t* = 0.535; *p* = 0.594; persistence: estimate = 0.006; SE = 0.003; *t *= 1.771; *p* = 0.079; dietary breadth: estimate = 0.035; SE = 0.026; *t* = 1.368; *p* = 0.174). We observed a tentative interaction between environmental predictability and sex for dietary breadth, with females in the unpredictable environment and males in the predictable environment showing a greater dietary breadth than males in the unpredictable environment and females in the predictable environment (Table 3). No significant interactions were observed between environmental predictability, sex and: IC; baseline worm latency; body condition; or colour learning (Table 3). Finally, of the 179 chicks that hatched, we found no evidence that participation, i.e. whether or not subjects interacted with the test apparatus and were included in the analyses, differed between the two environmental treatments (predictable environment: 66 participation, 26 non-participation; unpredictable environment: 51 participation, 36 non-participation: *χ*^2^ = 3.399; *p* = 0.065).

## Discussion

Environmental predictability was the strongest predictor of inhibitory control (IC) performance in pheasants. In contrast to our predictions, we found that birds reared in an unpredictable environment showed greater IC and were less persistent in their unrewarded attempts to acquire inaccessible mealworms, than birds reared in a predictable environment. IC performance did not differ between sexes and was unrelated to a suite of motivational measures, including latencies to acquire a freely available baseline worm, dietary breadth, persistence and body condition. Moreover, differences in IC were also unrelated to differences in a general learning ability that was assayed using a colour discrimination task. These effects did not differ between two different assays of IC, in which birds either had to reach inside a transparent cylinder or walk around a transparent barrier. Our findings show that, at least in pheasants, individuals that spend their first 8 weeks of life living under unpredictable environmental conditions exhibit enhanced IC performance on detour tasks, and that this relationship is not driven by changes in other motivational or cognitive processes that may be affected by the early life environment.

One explanation for our findings is that birds reared in an unpredictable environment were more reluctant to interact with a test apparatus. Accordingly, neophobic responses towards the test apparatus may have resulted in fewer pecks (errors), which were interpreted as greater IC. However, we consider this explanation unlikely as we found no differences in participation between the environmental treatments. Latencies to approach the IC test apparatus and acquire a freely available baseline worm also did not differ between the treatments. Moreover, there has been no evidence to suggest that neophobia influences IC performance in other species, such as dogs (Stow et al. 2018). A second explanation may be that because birds reared in the unpredictable environment were also less persistent in their attempts to obtain an inaccessible reward, they may have stopped pecking at the IC apparatus more readily. We would have attributed this lower level of pecking as better IC. We consider this explanation unlikely as, within treatments, persistence was not a strong predictor of IC performance. Reduced persistence may be indicative of altered motivation, yet we found little evidence that our other motivational measures differed between treatments or were good predictors of IC within treatments. A third explanation is that the different environmental treatments altered other cognitive processes, which may in-turn influence capacities for IC. Although we acknowledge that different environmental treatments may influence cognitive processes that we did not measure in the current study, we found no evidence that environmental predictability influenced pheasants’ learning performances on a visual discrimination task. Moreover, individual differences in learning performances failed to predict IC performance.

The type of IC task administered (barrier or cylinder) did not account for IC performance. Although performances were standardised (mean centred) across the two different tasks to account for differences in variance that may have arisen from differences in task difficulty, these findings suggest some consistency in our measures of IC. However, these findings are based on a between-subjects design, where individual measures of IC were reported from performances on either a barrier or a cylinder task, but not from both tasks. Although there is broad evidence of consistent (repeatable) individual performances across different cognitive tasks (Cauchoix et al. 2018), there is no evidence of consistent individual performances across different IC tasks (Brucks et al. 2017; van Horik et al. 2018a; Völter et al. 2018). Further investigation into the construct validly of different IC tasks therefore remains necessary.

Our finding, that early life environmental unpredictability improves capacities for IC in pheasants, contrasts with observations in humans. Humans exhibit poor IC after experiencing unpredictable conditions; either in terms of social interactions (Kidd et al. 2013; Michaelson et al. 2013), or environmental conditions (Frankenhuis et al. 2016). While the mechanisms underlying IC may differ between human and non-human animals, it remains difficult to interpret the adaptive advantage of high IC in pheasants reared in spatially unpredictable environments. It is possible that the contrasting influence of environmental predictability on IC between humans and pheasants is due to differences in the tasks used to measure IC and the associated processes of inhibition that regulate performance on these tasks. Studies that investigate the effects of unpredictable environmental conditions on IC in children typically use delay of gratification paradigms (Kidd et al. 2013; Michaelson et al. 2013), such as the marshmallow task (Mischel 1974), which require subjects to forgo an immediate reward for a larger reward in the future. Other measures of human IC include observational ratings from parents, teachers and self-reports of impulsive aggression, hyperactivity, lack of persistence, inattention and impulsivity (Moffitt et al. 2011). By contrast, pheasants in our study were required to inhibit their prepotent behavioural responses towards a desired food reward positioned behind a transparent barrier (i.e. response inhibition). Perhaps these different IC tasks (delayed gratification and motor inhibition) capture different processes of IC (Beran 2015), which in turn may also be influenced by different emotional, cognitive and behavioural states (Nigg 2017). Future studies may, therefore, benefit from investigating whether unpredictability also influences IC performance on delay of gratification paradigms that have been used successfully in other species of birds, such as corvids, *Corvus corone* and *C. corax*, (Dufour et al. 2012), Goffin cockatoos, *Cacatua goffini*, (Auersperg et al. 2013), pigeons, *Columba livia*, (Ainslie 1974) and domestic chicks, *Gallus gallus*, (Amita et al. 2010).

The contrasting influence of environmental predictability on IC between studies on humans and pheasants may also be attributable to differences in the experimental manipulations of predictability. In human studies, both Kidd and colleagues (2013) and Michaelson and colleagues (2013) manipulated environmental predictability socially. Kidd and colleagues (2013) manipulated the reliability of expected information, i.e. that an experimenter would return with crayons as promised, whereas Michaelson and colleagues (2013) altered perception of trust using images of “trustworthy” and “untrustworthy” faces. The manipulation of predictability on the development of IC could be further tested experimentally, by altering the predictability of non-social cues, such as previously learned contingencies involving food rewards, or social cues, such as the ‘trustworthiness” of different individuals using a cache retrieval paradigm (Dally et al. 2005).

Another explanation is that our manipulation may have altered environmental predictability, yet for birds reared under otherwise fairly mundane artificial husbandry, our intervention was not detrimental or stressful, but rather enriching and stimulating. Future studies may further address this by assaying proxies of stress, such as cortisol levels taken from saliva (acute measures) or faecal (chronic measures) samples, from each treatment group. In humans, a number of interventions, aimed at encouraging diverse activities that involve repeated practice and progressive challenges, such as computer games that increase working memory demands, have been found to enhance the development of IC in children (Diamond and Lee 2011). It may be possible that our movement of the partitions had analogous effects to those interventions that stimulate the development of IC in children. For example, repeated partition movement may have facilitated more diverse food-searching behaviours or encouraged exploration, which influenced working memory capacities and had corresponding influences on IC. Accordingly, environmental enrichment enhances IC in dogs (Clarke et al. 1951), whose IC may be bolstered by training programmes (Barrera et al. 2018) and more secure human interactions (Fagnani et al. 2016). However, we found no evidence to suggest that performances on the learning task differed between treatments. Future studies of non-human animals could investigate these relationships by including additional cognitive and behavioural assays of individuals in predictable and unpredictable environmental conditions, such as exploring foraging and movement strategies and the associated payoffs for individuals in each environment.

Our findings support a growing consensus that an individual’s performance on detour tasks may be highly susceptible to different testing conditions. We found that environmental unpredictability enhanced IC performances of pheasants on transparent detour tasks, and that these differences could not be explained by various motivational measures or general processes of discrimination learning. While we acknowledge the ease in which detour tasks can be administered to a variety of species, we highlight the importance of considering an individual’s developmental experience when inferring capacities of IC.
